# Optimization of decellularized human placental macroporous scaffolds for spermatogonial stem cells homing

**DOI:** 10.1007/s10856-021-06517-7

**Published:** 2021-04-23

**Authors:** Fatemeh Asgari, Hamid Reza Asgari, Mohammad Najafi, Behnaz Sadat Eftekhari, Mina Vardiani, Mazaher Gholipourmalekabadi, Morteza Koruji

**Affiliations:** 1 Stem Cell and Regenerative Medicine Research Center, Iran University of Medical Sciences, Tehran, Iran; 2 Department of Anatomical Sciences, School of Medicine, Iran University of Medical Sciences, Tehran, Iran; 3 Biochemistry Department, Iran University of Medical Sciences, Tehran, Iran; 4 Cellular and Molecular Research Centre, Iran University of Medicine Sciences, Tehran, Iran; 5 Biomaterials Group, Faculty of Biomedical Engineering, Amirkabir University of Technology, Tehran, Iran; 6 Department of Physiology and Institute for Medicine and Engineering, University of Pennsylvania, Philadelphia, USA; 7 Reproductive Biotechnology Research Center, Avicenna Research Institute, ACECR, Tehran, Iran, Tehran, Iran; 8 Department of Tissue Engineering & Regenerative Medicine, Faculty of Advanced Technologies in Medicine, Iran University of Medical Sciences, Tehran, Iran; 9 Department of Medical Biotechnology, Faculty of Allied Medicine, Iran University of Medical Sciences, Tehran, Iran

## Abstract

Decellularized scaffolds have been found to be excellent platforms for tissue engineering applications. The attempts are still being made to optimize a decellularization protocol with successful removal of the cells with minimal damages to extracellular matrix components. We examined twelve decellularization procedures using different concentrations of Sodium dodecyl sulfate and Triton X-100 (alone or in combination), and incubation time points of 15 or 30 min. Then, the potential of the decellularized scaffold as a three-dimensional substrate for colony formation capacity of mouse spermatogonial stem cells was determined. The morphological, degradation, biocompatibility, and swelling properties of the samples were fully characterized. The 0.5%/30 SDS/Triton showed optimal decellularization with minimal negative effects on ECM (*P* ≤ 0.05). The swelling ratios increased with the increase of SDS and Triton concentration and incubation time. Only 0.5%/15 and 30 SDS showed a significant decrease in the SSCs viability compared with other groups (*P* < 0.05). The SSCs colony formation was clearly observed under SEM and H&E stained slides. The cells infiltrated into the subcutaneously implanted scaffold at days 7 and 30 post-implantation with no sign of graft rejection. Our data suggest the %0.5/30 SDS/Triton as an excellent platform for tissue engineering and reproductive biology applications.

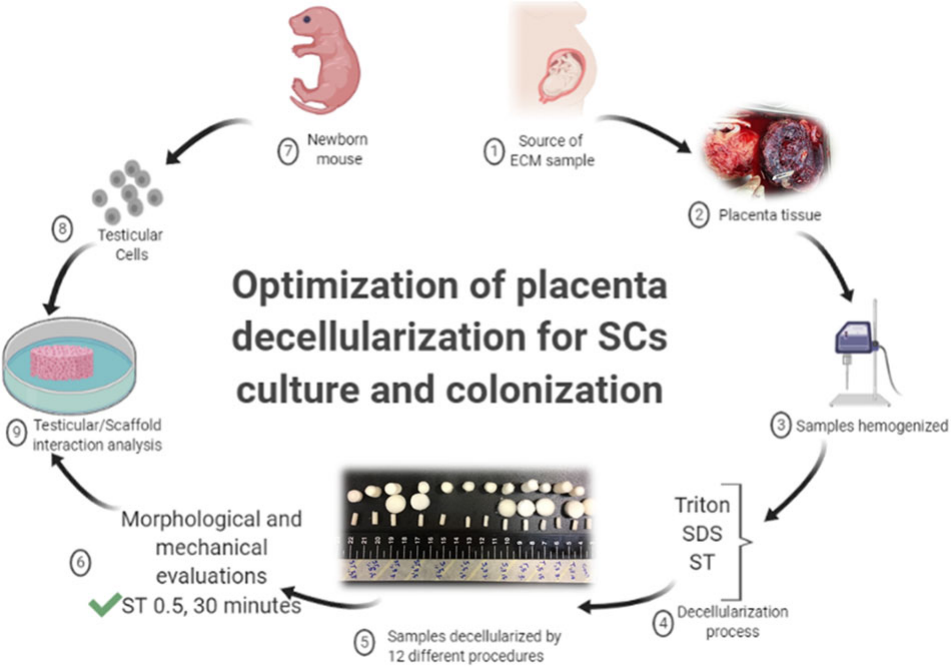

## Introduction

About 15% of couples have infertility problems, half of which are related to men [1]. In recent years, many techniques have improved fertility in men [2, 3]. Therefore, after a century of studies of spermatogenesis in different species, it is still a good way to treat infertility in people with congenital testicular abnormalities (Klein–Filter syndrome, cryptorchidism, and androgen insensitivity syndrome), azoospermic men who do not have spermatids, and also, there are no immature patients with cancer who have suffered spermatogenic cell damage during chemotherapy and radiotherapy [4]. This low population of spermatogonia in mice is responsible for producing 10^9^ sperm per day [5]. Therefore, the aim of the researchers was to investigate the proliferation and differentiation of spermatogonia to produce functional sperm in vitro [6]. In this regard, a wide range of 3-dimensional (3-D) tissue engineering scaffolds have been made from synthetic and biological materials, each of which has its advantages and disadvantages [7, 8]. Scaffolds place the cells in contact with nutrients and oxygen, and facilitate the disposal of waste materials [9]. Tissue engineering scaffolds can provide a 3-D microstructure with tunable biomechanical and biological properties to achieve a biomimetic substrate for treatment of damaged tissues such as skin [10], cartilage [11], eye [12], or specific lineage differentiation of stem cells both in vitro and in vivo [13].

Decellularization of tissue and production of engineering scaffolds from extracellular matrix (ECM) components are considered as an excellent biomedical platform that provides a normal tissue biomimetic micro/macrostructure with many biological factors required for cell migration, proliferation, and further conduction/promoting differentiation [14–16]. To date, several decellularization protocols have been examined for successful and safe decellularization of tissues. It is well documented that decellularization and preservation processes profoundly affect the biomechanical and biological properties of tissue. Therefore, the intensive efforts are still being made to find an optimized and safe decellularization and preservation methods with full removal of cells and cell fragments with minimal damage to ECM [17, 18].

In the current study, we aim to optimize the decellularization of human placenta using different decellularization agents and time points for tissue engineering and reproduction sciences applications. Placenta is excreted after birth, and so is easily available with a large amount of ECM. Placenta is an excellent source of biological materials for production of allograft scaffolds [19, 20]. One of the advantages of using placenta of tissue is the lack of an invasive method for tissue acquisition [21]. ECM of placenta contains various fibers including fibronectin, laminin, and collagen type I, III, IV, V, and VI [22]. It is also a rich reservoir for different types of growth factors and cytokines such as epidermal growth factor (EGF), transforming growth factor (TGF-β, β, fibroblast growth factor (FGF), platelet-derived growth factor (PDGF), and vascular endothelial growth factor (VEGF) [23].

Almost all decellularization procedures are detergent- (ionic and anionic-based detergents), freeze/thawed- and mechanical-based protocols. Sodium dodecyl sulfate (SDS, as anionic detergent) and Triton X100 (as nonionic detergent) have widely been used for decellularization of various tissues. It is reported that SDS is the most effective detergent for removal of the cells from tissues. However, SDS also showed to have toxicity properties against human cells and also causes extensive damages to tissues, dependent to its concentration and incubation time point [18, 19].

Here, we examine twelve decellularization protocols using different concentrations of SDS and Triton X100 and treatment time points for decellularization of human placenta and fabrication of placental-derived macroporous scaffolds with 3-D microstructure. The successful decellularization and damages to ECM are investigated with DNA count and various tissue staining. The morphological, biomechanical, biochemical, and biological properties of the scaffold are fully characterized in vitro and in vivo. The potential of the optimized placental macroporous scaffold for culture, growth, attachment, and colony formation of SSCs are then determined.

## Materials and methods

In this study, we compared different protocols of decellularization of human placenta, and fully characterized the decellularization, mechanical and biological properties. The potential of the acellular 3-D sponge placenta scaffolds for SSCs culture and growth was studied in vitro.

### Tissue collection

The study was conducted under the Declaration of Helsinki. Five human placentas were obtained from Akbar-Abadi, Hospital in Tehran and after ethical approval the informed consent from cesarean deliveries at the Iran University of medical sciences was taken (Approval ID: IR.IUMS.FMD.REC 1396.33110).

### Decellularization procedures

In the first step, the placenta was washed with distilled water for separating the blood clots [24]. The amniotic and chorionic membranes and umbilical cord were completely isolated and removed and considered tissue was split into small pieces as much as possible and homogenized for 10 min on the ice using a blender. The homogenized placenta washed by distilled water and centrifuged at 1500 RPM for 10 min. The samples were treated with different concentrations of Triton (Triton X100, 9002-93-1, Sigma-Aldrich) and SDS (sodium dodecyl sulfate, Sigma-Aldrich) for 15 or 30 min, in twelve decellularization groups (Table 1). After decellularization, the samples were rinsed with distilled water. The treated subgroups of ECM were centrifuged at 2000 RPM and rinsed with distilled water two times for 8 days in a cold room on the shaker, until the extraction of detergents. ECM was gently poured into 24 wells, frozen at –80 °C, and dried (Alpha 1–2 LD plus, Christ, Germany) overnight. All the decellularized scaffolds were kept at −80 °C until use, maximum for 1 month. The process of placenta decellularization was done in three replications. Decellularized ECM sheets were washed 4 times by PBS and penicillin (300 IU/mL), streptomycin (300 μg/mL), every 15 min, and sterilized by UV before SSCs in vitro culture.

**Table 1:** Different procedures used for decellularization of human placenta tissue in this study

Decellularization agent	Concentration (w/v %)	Exposure time (min)
No treatment (control)	No treatment	–
Triton X-100	0.3	15
	0.3	30
	0.5	15
	0.5	30
SDS	0.3	15
	0.3	30
	0.5	15
	0.5	30
SDS + Triton X-100	0.3	15
	0.3	30
	0.5	15
	0.5	30

### Characterizations

#### H&E and DAPI staining

The samples were fixed with 10% formalin, dehydrated through a graded series of alcohol; 50, 70, 80, 95, and 3 × 100%, embedded in paraffin, and cut at 5 µm thickness by a microtome. The slides were stained with hematoxylin and eosin (H&E) and 4′, 6-diamidino-2-phenylindole (DAPI, Thermo Scientific). The H&E and DAPI stained slides were visualized under a light microscope (Olympus, Japan) at ×400 magnifications using an Olympus DP72 digital camera, and a fluorescence microscope, respectively. Cell nuclei in H&E and DAPI staining are stained in blue. The cell’s nucleus of the samples was measured in 10 random fields and compared between the decellularization groups.

#### DNA content

To quantify the DNA content in native and decellularized tissues, samples were lyophilized and then the extraction of DNA was done from ~7 mg of dry samples by the QiaAmp mini kit (Qiagen, USA) according to manufacturer’s protocol. Finally, the NanoDrop spectrophotometer (2000C, Thermo Fisher Scientific, USA) used to determine the concentration of the total DNA (ng/mg).

#### Orcein, alcian blue, and Masson’s trichrome

The sectioned slides were stained with Masson’s trichrome (Sigma-Aldrich, USA), 1% Alcian Blue (diluted in 0.1 M HCl, Sigma-Aldrich, USA), and Orcein (Taenzer-Unna) for visualization of collagen fibers, glycosaminoglycan (GAG), and elastic fibers, respectively. All techniques were performed in accordance with the manufacturer’s instructions (*n* = 3 per condition, from all treated placenta). Images were captured on ten random fields by a light microscope and analyzed by program ImageJ (US National Institute of Health, Bethesda, MD).

#### Mechanical behavior

The mechanical properties of the decellularized samples were measured using a Universal Testing Machine (Hct400/25, Zwick/Roell) with a 10 kg load cell and a crosshead loading rate of 0.5 mm.min^−1^. The cylindrical samples presented for each type of decellularized method were all 4 mm in diameter and 8 mm in height. The obtained strain-strain curves were analyzed for measurement of the mechanical parameters such as compressive strength and Young’s modulus. The ultimate compressive strength of materials was specified as value of uniaxial compressive stress, which broke the sample. By calculating the ratio of stress to strain in the linear elastic region, the Young’s modulus was determined.

#### Degradability assays

The in vitro degradation assay of cylindrical decellularized scaffolds was performed for three samples from each group with the same weight, which were soaked into 5 ml of PBS at pH = 7.4 and 37 °C. At a predetermined time point (3, 7, 10, 14, 21, and 30 days), each of the samples was taken out and was freeze-dried for 12 h after surface wiping and their weight recorded. Water absorption and sample weight loss were calculated by Eqs. (1) and (2), respectively:

1 $${\mathrm{Water}}\,{\mathrm{absorption}}\,\left( \% \right):\left( {{\mathrm{W}}_{\mathrm{t}} - {\mathrm{W}}_{\left. 0 \right)/}{\mathrm{W}}_0 \times 100\% } \right.$$

2 $${\mathrm{Degradation}}\,\left( \% \right):\left( {{\mathrm{W}}_0 - {\mathrm{W}}_{\mathrm{d}}} \right)/{\mathrm{W}}_0 \times 100\%$$

where *W*_0_ is the initial dry weight, *W*_d_ is the dry sample weight after removal from the medium, and *W*_t_ is the wet sample weight after removal. Furthermore, pH values of the solutions during scaffold soaking were recorded.

#### The swelling behavior

The equilibrated swelling ratio (ESR) of each type of decellularized scaffolds was measured using gravimetric method. The cylindrical sample with same size was immersed in PBS at 37 °C for 24 h. After reaching the swelling equilibrium, the scaffolds were removed from the buffer solution, the excess water of sample was removed by filter paper and swollen samples were weighed. The ESR was calculated by following equation:

$${\mathrm{ESR}} = \left( {{\mathrm{Ws}} - {\mathrm{W}}0} \right)/{\mathrm{W}}0 \times 100\%$$

In this equation, Ws and W0 parameters represent the stolen and initial weight of scaffold, respectively. The experiments were carried out for three samples of each group.

### Microstructure of scaffold

#### Scanning electron microscopy

First, we coated fixed samples with gold [25]; then, their morphology was explored using Scanning Electron Microscopy (SEM, AIS2100; Seron Technology, Gyeonggi-do, South Korea).

#### Pore size

The average diameters of pore size in the samples was measured from ten random fields of SEM micrographs (300x magnification) using image analyzing program ImageJ (US National Institute of Health, Bethesda, MD).

### In vitro SSCs-scaffold interaction

#### SSCs isolation and cell culture

Ten 3–6-day-old male mice from the National Medical Research Institute, initially from the original stocks of Razi Laboratory (Tehran, Iran), were used in the experiment. The animals were kept in cages at a temperature of 25 °C, with a 12-h light and dark cycle. The animals had free access to drinking water and standard laboratory pellets. The research was conducted in accordance with the National Research Council guidelines. Testes were isolated and randomly assigned to two experimental groups: two dimensional (control) and three-dimensional (experiment) for in vitro SSCs cultured for MTT, SEM, and H&E assessment on scaffold.

The testes were collected and suspended in PBS supplemented with penicillin (100 IU/mL), streptomycin (100 μg/m). SSCs were isolated based on the method described previously [26]. Briefly, the testes were placed in Dulbecco’s Modified Eagle medium (DMEM/F12) including 0.5 mg/mL collagenase ІV (Sigma-Aldrich), 0.5 mg/mL Trypsin (Sigma-Aldrich), and 0.05 mg/mL DNAse (Sigma-Aldrich, USA), for 30 min with pipetting and stay at 37 °C. The interstitial cells were removed by washing in DMEM/F12 medium and it was centrifuged. Plates of cells extracted and second digestion step was done according to previous step. Then seminiferous cord fragments removed and cells extracted by centrifuging.

Primary culture of SSCs was performed in DMEM/F12 supplemented with 10 ng/mL GDNF (Glial cell line-derived neurotrophic factor, RP-1107, Royan Institute) and 2% FBS in (FBS, Gibco), 1 % penicillin-streptomycin (15140-148, Gibco), and 1% non-essential amino acids (11140-035, Gibco) for one week. The incubation of the cells was done at 35 °C, 5% CO_2_ in a humidified atmosphere, and the medium was replaced three times. After 1 week, the mix of SSCs was used for cells and scaffold interaction analysis. Prior to culturing on the optimized scaffold, the cell number was determined by a hematocytometer. The SSCs viability was also evaluated by the dye exclusion test (0.04% trypan blue solution).

#### Identification of SCs by RT-PCR

The isolated SCs was identified by amplification of SSCs specific genes (*Gfrα1* (*GDNF* family co-receptor α1), *Plzf* (promyelocytic leukemia zinc-finger), *Id4* (Inhibitor of DNA Binding 4) and Gapdh genes, as housekeeping gene) by reverse transcription-polymerase chain reaction (*RT-PCR*). Primers were designed using Gene Runner software (version 3.02; Hastings Software Inc, New York, NY, USA) and online NCBI primer design software (ncbi.nlm.nih.gov/tools/primer-blast/). Table 2 shows list of primers used in this study. Total RNA was extracted using a standard RNA extraction kit (Qiagen, Hilden, Germany), according to the manufacturer’s instructions, and confirmed by a 260/280 nm optical density absorbance ratio measurement. 1 μL of PCR products for each amplicon were run in a 1.2% agarose gel embedded in Tris-Borate-EDTA (TBE) 1× loading buffer (Sigma-Aldrich) at a voltage of 95 for 45 min. The gels were stained with 0.1 μg/mL Gel Red™ (Biotium Inc, Hayward, CA, USA) and the bands were visualized using Gel Logic (Carestream Health Inc., Rochester, NY, USA) and images were obtained [7].

**Table 2:** Sequences of primers designed for Identification of SCs by reverse transcription-polymerase chain reaction (RT-PCR)

Gene name	bp	Primers sequences	Melt (°C)
Plzf	137	F: CCCGTTGGGGGTCAGCTAGA	61
		R: CTGCAAGGTGGGGCGGTGTAG	
Gfrα1	130	F: CTGTGGACTAGCTCGCTCTC	60
		R: GACCCGCTTTTAGGGGTTCA	
Id4	185	F: GGGTGACAGCATTCTCTGC	58.52
		R: TTGGAATGACAAGACGAGACG	
Gapdh	125	F: CTGCTGGACAAGTGAGTCCC	60
		R: CCAAGTACCCTGGCCTCATC	

#### Cell proliferation and attachment

The scaffolds in the size of 2 × 5 × 5 mm^3^ were washed with PBS and were incubated with a culture medium at 35 °C overnight prior to use. The scaffolds were placed in a 24-well plate, and seeded with 1 × 10^4^ number of SSCs. The cells-scaffold constructs were incubated in a cell culture incubator with standard atmosphere at 35 °C for 1, 3, and 7 days. mitochondrial activity for reduction of MTT salt [27]. The optical density for each sample, representing the cell viability value, was measured for each sample using a microplate ELISA reader at a wavelength of 570 nm with a reference filter of 620 nm. The cell cultured in plastic surface of cell culture plated and medium alone served as control (100% cell viability).

For assessment of cells-scaffold interaction, the 1 × 10^6^ SSCs were seeded on 1 cm of the scaffold, and then incubated in 5% CO_2_ for one week at 35 °C. The cell-scaffold constructs were fixed with 2.5% glutaraldehyde (Merck) for 2 h and dehydrated in a graded concentration (30, 50, 70, and 100%) of alcohol, and then were lyophilized overnight. The samples were sputter-coated with gold and observed by electron microscope (SEM, AIS2100; Seron Technology, South Korea) at an accelerating voltage of 15 kV.

#### SSCs colony formation

The optimized decellularized scaffold was investigated for its potential as a substrate for colony formation of SSCs. For visualization of colony formation, the re-cellularized scaffold after 7 days cell culture incubation time were fixed with 10% formalin, dehydrated through a graded series of alcohol, embedded in paraffin, and sectioned at 5 µm, as described above. The samples were stained with H&E and formation of SSCs colony was observed and investigated under light microscope.

### In vivo biocompatibility assay

In vivo biocompatibility assay was carried out by a procedure described in our previously published article [28]. For in vivo biocompatibility assay, six NMRI male mice (6–8 week, 30 g) were obtained from the National Medical Research Institute, initially from the original stocks of Razi Laboratory (Tehran, Iran). Anesthesia was done by intra-peritoneal injection of ketamine (0.1 mg/kg, Anesketin, Heusden–Zolder, Belgium) and xylazine (0.01 mg/kg, Heusden–Zolder, Belgium). After fixing of mice on a special table, the operating site was shaved and sterilized with betadine 10% (Povidone Iodine 10%, Najo, Iran). The scaffolds in the size of 2 × 5 × 5 mm^3^ were washed with PBS and exposed to UV rays for 20 min and implanted subcutaneously on the back of mice. After surgical incision and scaffold transplantation, the skin of the surgical site was sutured with a single absorbable zero silk thread (Supasil 0.1, Supa, Iran). The animals were kept in cages at a temperature of 25 °C, with a 12-h light and dark cycle in pre-implantation and post-implantation period. The animals had free access to drinking water and standard laboratory pellets. Short term (7 days) and long term (30 days) in vivo biocompatibility of the optimized decellularized scaffold was determined. The mice were sacrifice by cervical dislocation and implanted tissue was collected and stained with H&E (*n* = 6, three samples for day 7 and three samples for day 30). The stained samples were observed under light microscope. Images were captured on 10 random fields by a light microscope and analyzed by program ImageJ (US National Institute of Health, Bethesda, MD). The macrophages, lymphocytes, and fibroblasts cells infiltrated into the implanted site were investigated.

### Statistical analysis

In the present study, the data analysis was conducted using Prism 7 and SPSS software for Windows, version 16.0 (SPSS Inc., Chicago, IL, USA). Variables were presented as the mean ± standard deviation. The analysis of variance followed by one way ANOVA and Tukey’s post-hoc was used for multiple group analyses. The *p* values of < 0.05 were set as significant in statistical terms.

## Result

### Characterizations

#### Decellularization confirmation

**Fig. 1:**
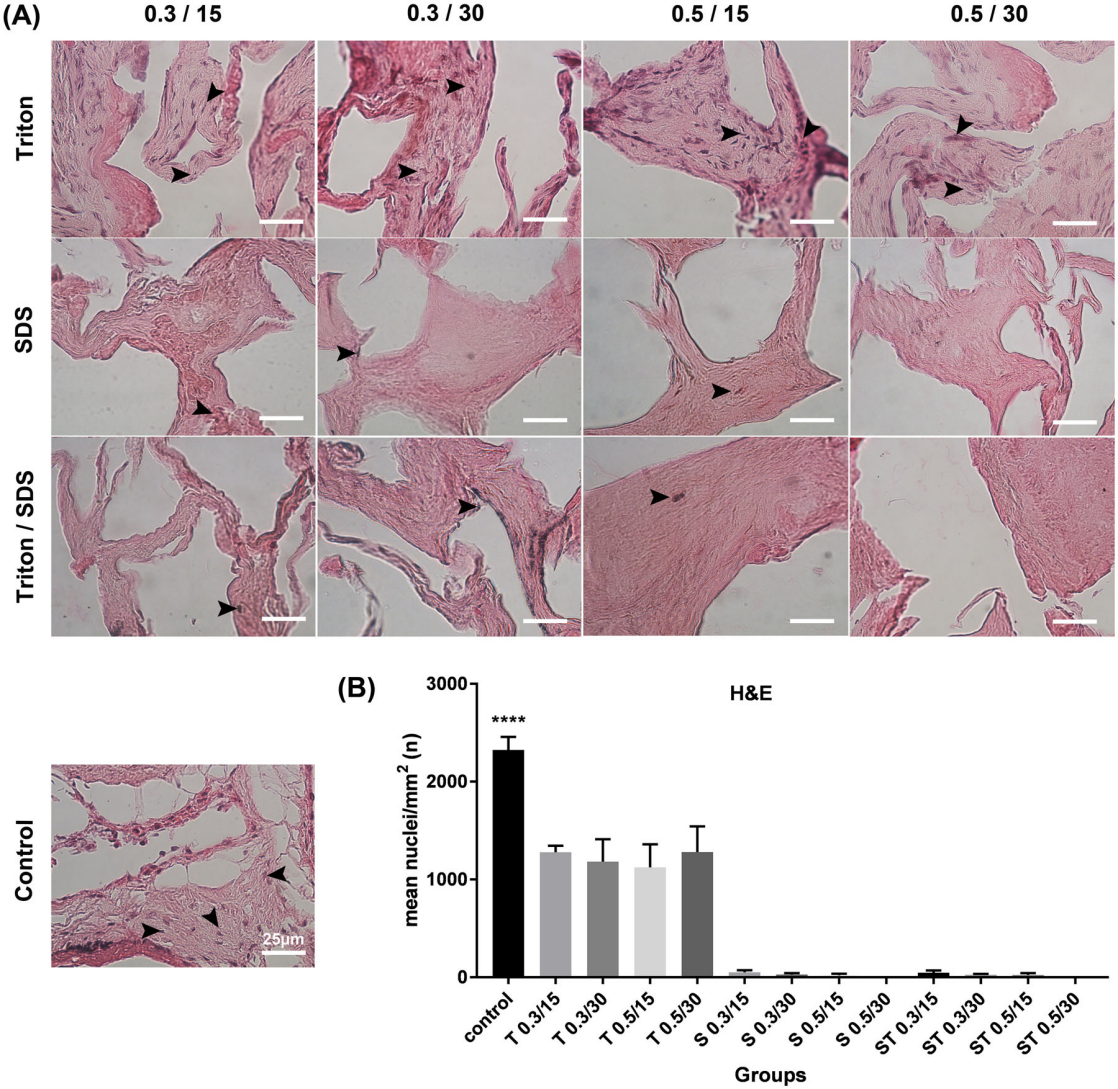
**A** Histological analysis of decellularized placenta fragments in fresh and experimental groups. **B** The number of nuclei decreased significantly in all of the decellularization protocols. The cells nuclei are shown with black arrows. Data are expressed as the mean ± SD, scale bar in all groups is: 25 µm (****P < 0.0001)

**Fig. 2:**
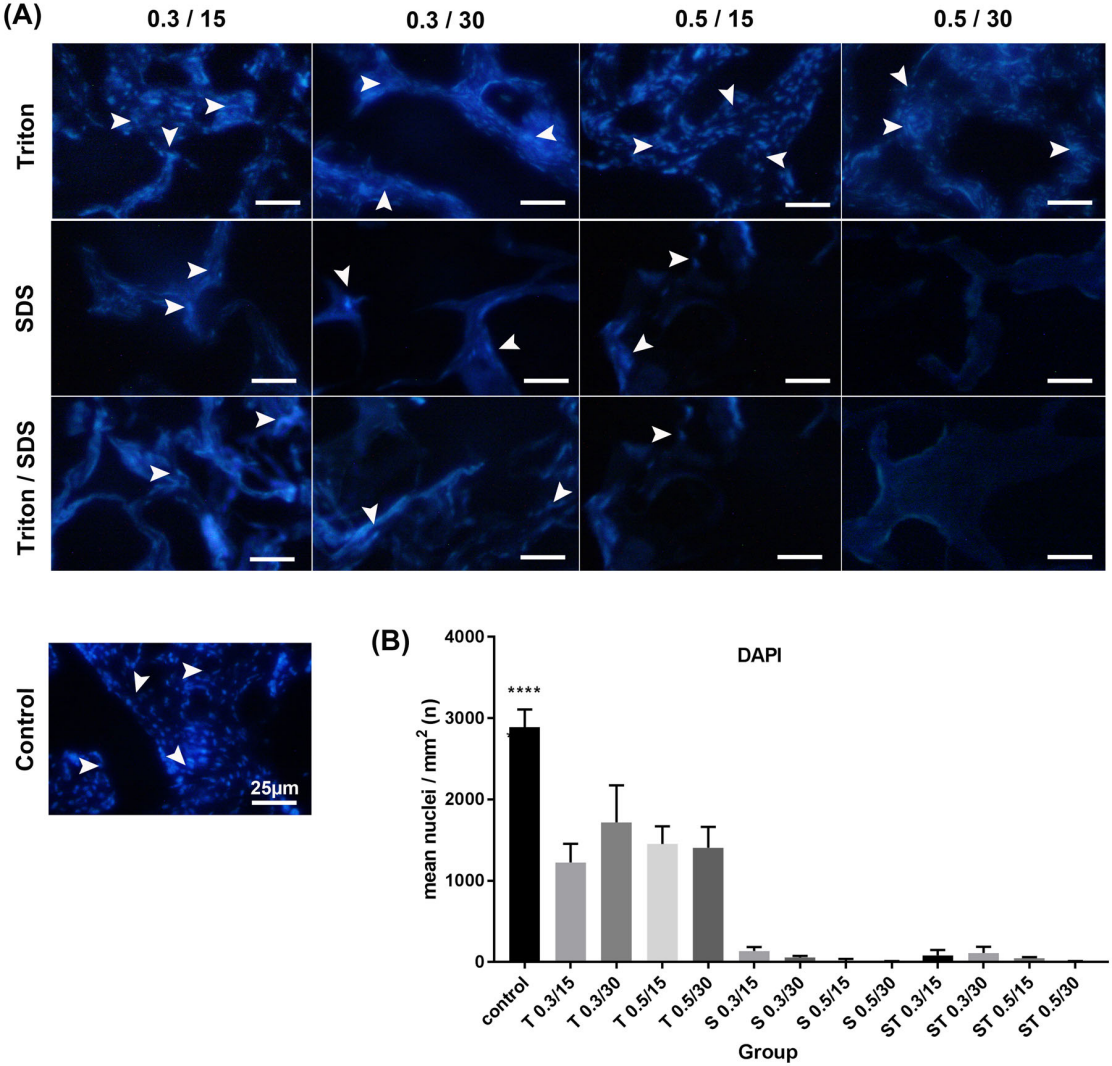
**A** DAPI staining in decellularized placenta fragments in fresh and experimental groups. **B** The number of nuclei decreased considerably in all of the decellularization protocols. The cells nuclei are shown with white arrows. Data are expressed as the mean ± SD, scale bar in all groups is: 25 µm (****P < 0.0001)

The successful removal of the cells and nucleic acids from the ECM was verified using H&E, DAPI staining, and DNA quantification. All of the decellularization treatments considerably reduced the DNA content in comparison with the native placenta samples. The dsDNA in ECM of ST 0.5 for 30 min and SDS 0.5 for 30 min was also quantified and compared to the native human placenta, respectively (28.49 ± 1.78, 33.32 ± 2.2 and 1476 ± 218 ng/mg, mean ± SD, *P* ≤ 0.05). The tissue denuded with ST 0.5 for 30 min and SDS 0.5 for 30 min detergents observed 1.99 and 2.25% of the DNA remained in tissue fragments after the decellularization process, respectively (Supplementary Fig. 1). The numbers of nuclei/mm^2^ after H&E staining significantly decreased after decellularization in all groups compared with native tissue (*P* ≤ 0.05) (Fig. 1A, B). The tissue treated with ST 0.5 for 30 min and SDS 0.5 for 30 min detergents showed lowest numbers of nuclei respectively (0.25 ± 0.5, 0.75 ± 0.95 nuclei/mm^2^, mean ± standard deviation (SD)) compared with native tissue (2324 ± 133 nuclei/mm^2^, *P* ≤ 0.05, Fig. 1B). Moreover, the number of nuclei/mm^2^ after DAPI staining confirmed the H&E results, which significantly decreased in treated groups compared with control group (*P* ≤ 0.05, Fig. 2B). The data obtained from DAPI staining revealed that the tissue treated with ST 0.5 for 30 min and SDS 0.5 for 30 min detergents observed lowest numbers of nuclei respectively (3.5 ± 7.0, 3.8 ± 7.5 nuclei/mm^2,^ mean ± SD) compared with control samples (2888 ± 219.6 nuclei/ mm^2^, *P* ≤ 0.05, Fig. 2B).

#### Extracellular matrix components

**Fig. 3:**
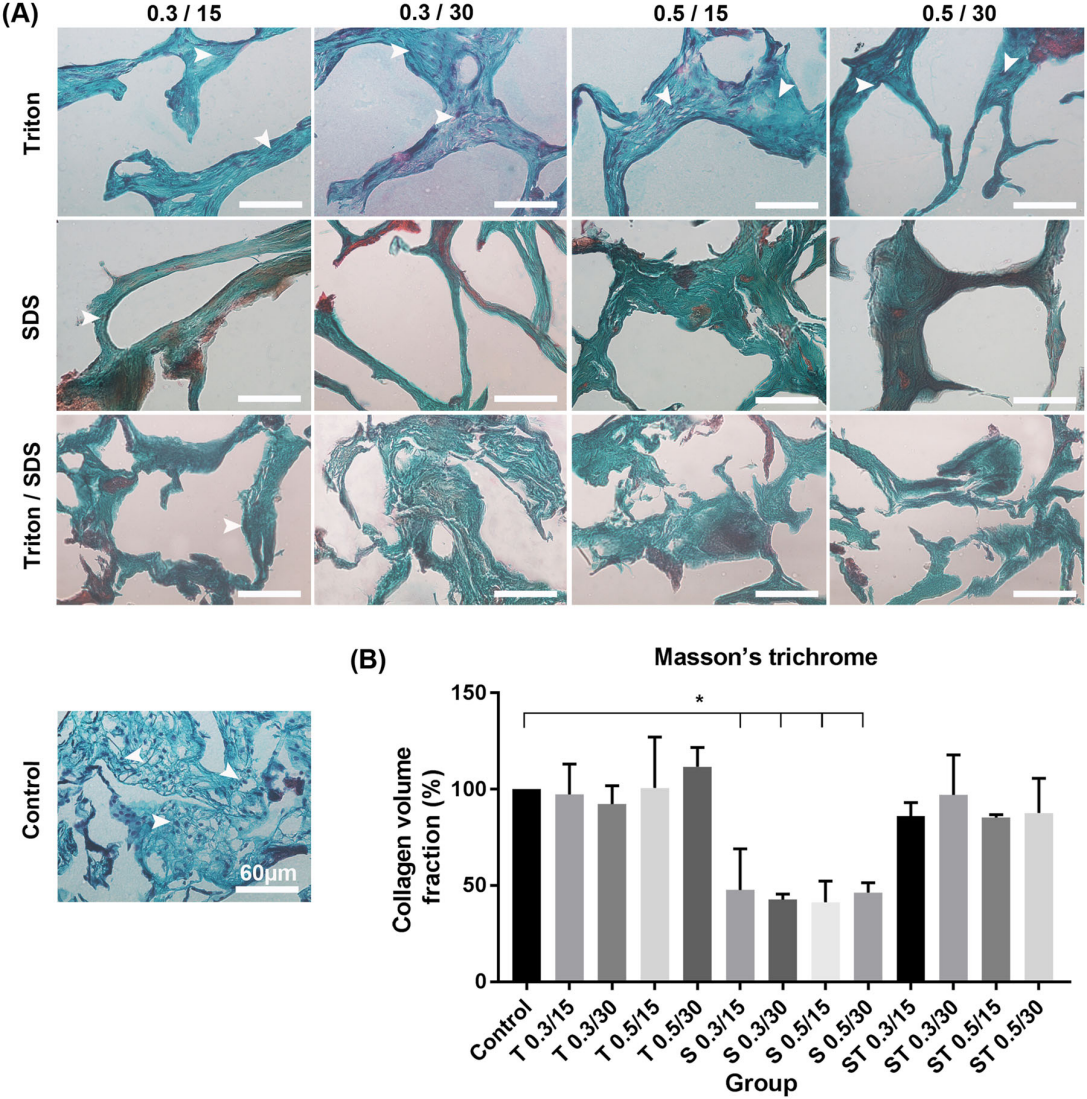
**A** Characterization of extracellular matrix components for recognition of collagen in the native and experimental tissue fragments by Masson’s trichrome staining. **B** The quantification of collagen revealed that SDS reduced collagen. The cell’s nuclei are shown by white arrows. All data are expressed as the mean/percentage ± SD, scale bar: 60 µm (*P < 0.05)

**Fig. 4:**
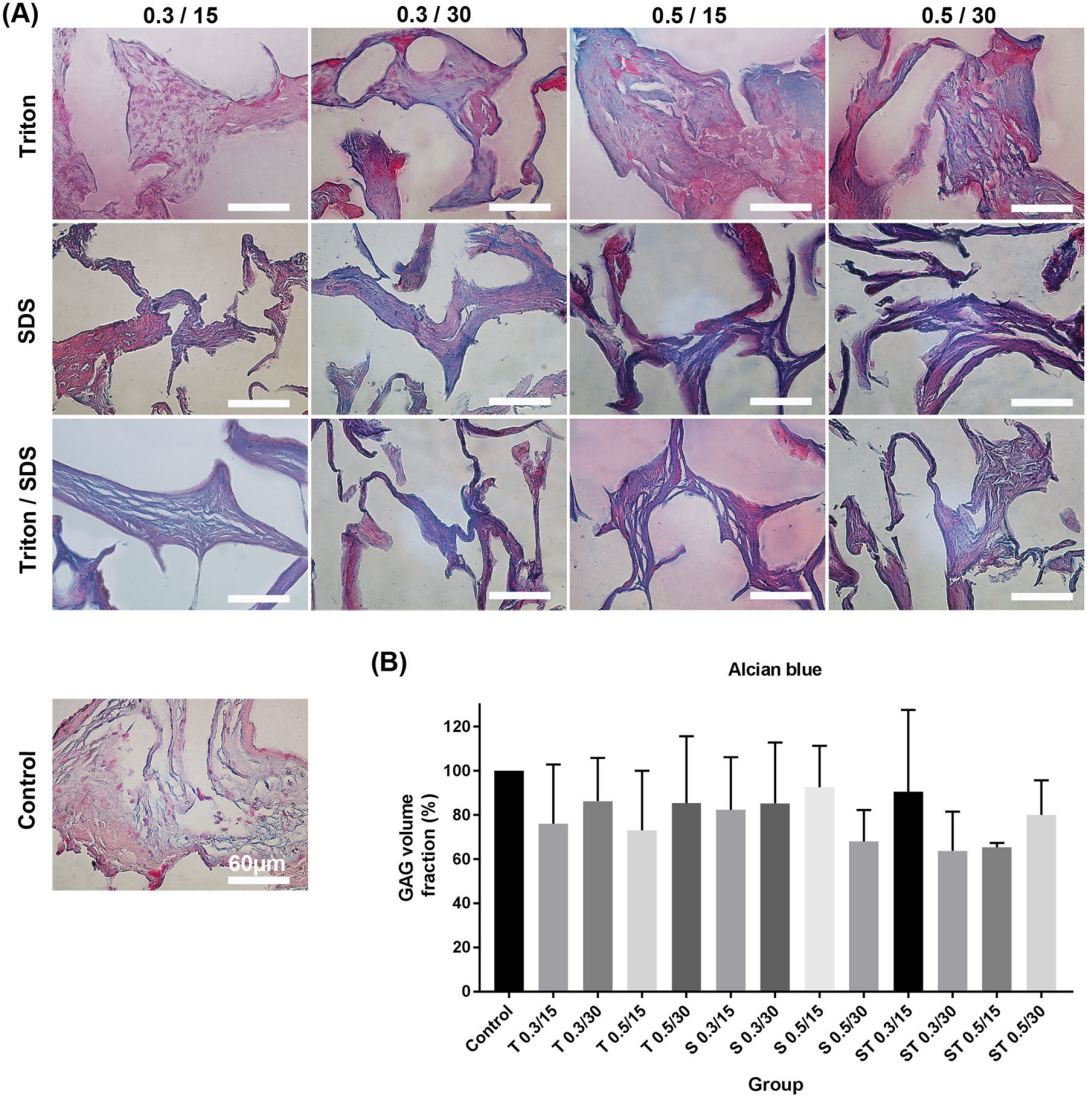
**A** Characterization of extracellular matrix components for detection of GAG in the native and experimental tissue fragments by Alcian blue staining. **B** There were no differences between groups compared to native tissue. All data are expressed as the mean/percentage ± SD, scale bar: 60 µm (*P* > 0.05)

**Fig. 5:**
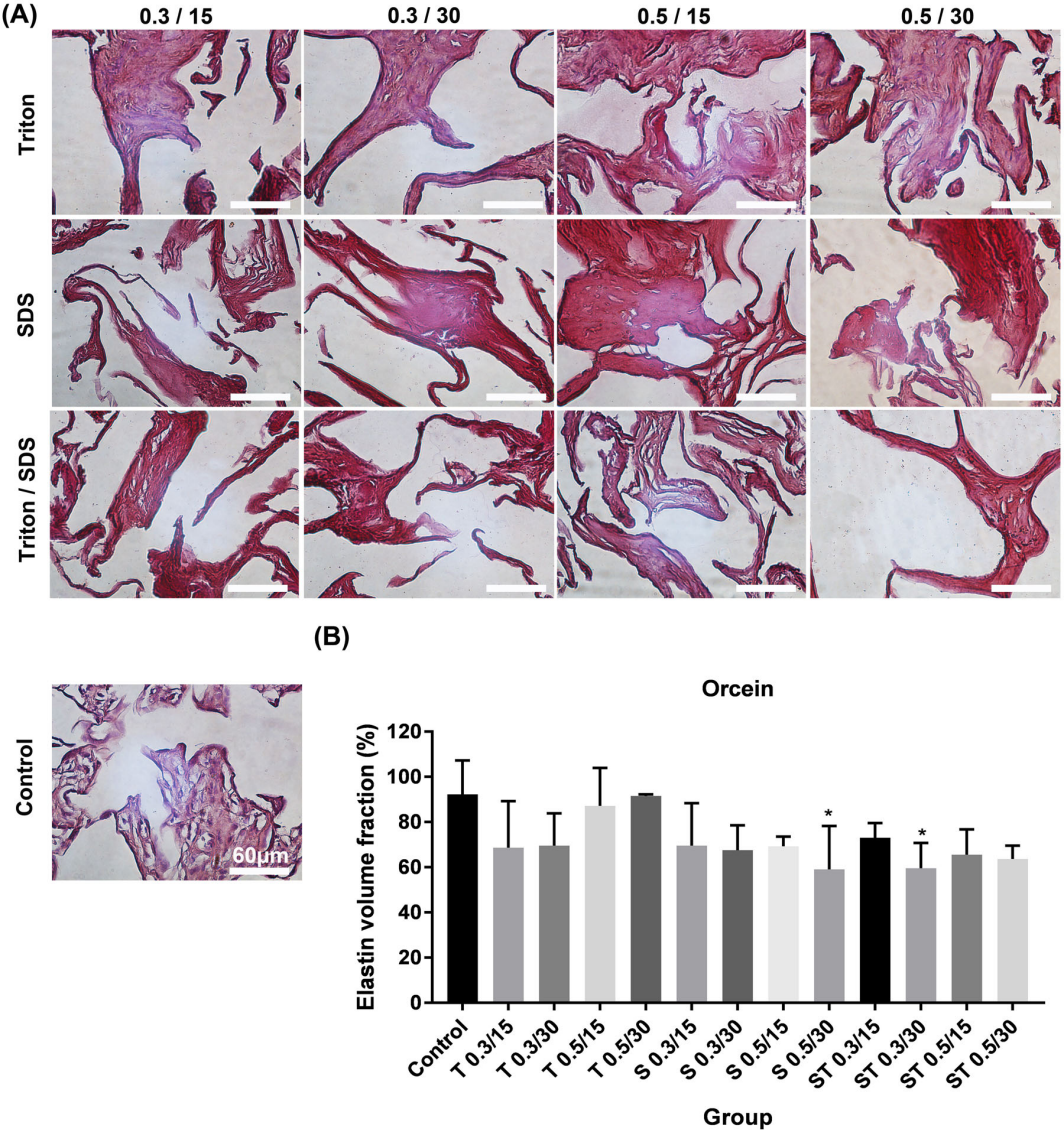
**A** Characterization of extracellular matrix components for detection of elastin in the native and experimental tissue fragments by Orcein staining. **B** All data are expressed as the mean/percentage ± SD, scale bar: 60 µm (*P > 0.05)

Placental ECM components such as collagen, elastin, and GAGs were visualized with Alcian blue, Orcein, and Masson’s trichrome staining, respectively, to determine the effects of each decellularization protocol on ECM damage. The results indicated that collagen component decreased in the tissues after treatment with SDS (*P* ≤ 0.05). The tissues decellularized with SDS 0.5% for 30 min and ST 0.3% for 30 min showed a slight decrease in elastin fibers and GAG compared with other groups (Figs. 3–5, *P* ≤ 0.05).

#### Mechanical behavior

As shown in Table 3, the SDS groups indicated the lowest ultimate stress and ultimate strain in comparison with other samples. Also, the decrease in elastic modulus of the samples was as follow: control > T samples > ST samples > SDS samples. The lowest percentages of ultimate strain observed in SDS samples.

**Table 3:** Mechanical properties of the decellularized samples

Group	Ultimate stress (MPa)	Ultimate strain (%)	Elastic modulus (MPa) (Young’s Modulus)
Control 1	5.44 ± 0.65	0.38 ± 0.04	34.35 ± 5.33
T3–15	6.96 ± 0.23	0.51 ± 0.02	31.32 ± 1.43
T3–30	6.52 ± 0.15	0.48 ± 0.07	30.12 ± 2.54
T5–15	6.13 ± 0.74	0.45 ± 0.06	28.63 ± 4.29
T5–30	5.94 ± 0.52	0.41 ± 0.01	27.43 ± 5.41
S3–15	3.97 ± 0.91	0.25 ± 0.06	18.121 ± 4.32
S3–30	3.51 ± 0.23	0.21 ± 0.01	16.64 ± 7.12
S5–15	2.91 ± 0.12	0.18 ± 0.05	14.23 ± 4.32
S5–30	2.51 ± 0.43	0.16 ± 0.06	13.56 ± 8.32
ST3–15	5.16 ± 0.38	0.38 ± 0.02	25.23 ± 1.39
ST3–30	4.95 ± 0.71	0.35 ± −0.030	22.23 ± 4.82
ST5–15	4.51 ± 0.39	0.31 ± 0.05	21.12 ± 5.43
ST5–30	4.29 ± 0.65	0.29 ± 0.04	19.84 ± 3.54

Mechanical tensile data are expressed as means ± SD, *n* = 3

#### Swelling and degradability behavior assays

**Fig. 6:**
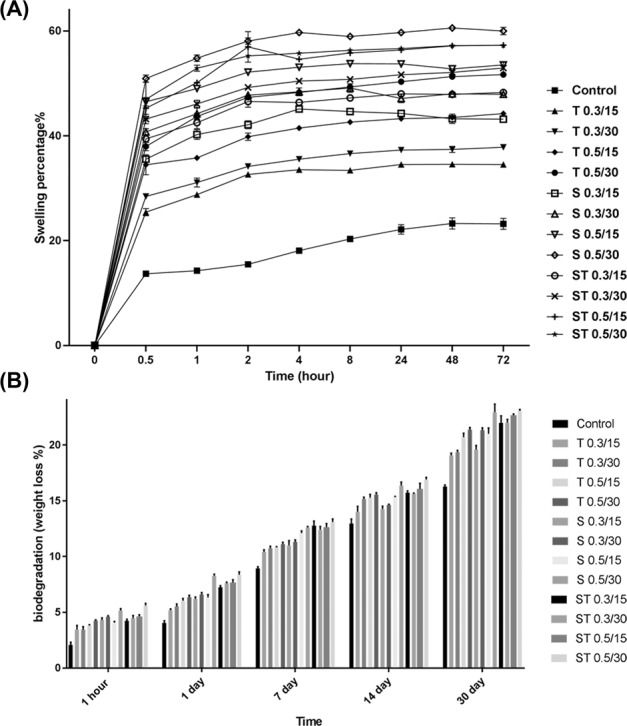
Swelling and degradability behavior assays. **A** The equilibrated swelling ratio of each type of decellullarized scaffolds. **B** The in vitro degradation assay of decellullarized scaffolds. Data are expressed as means ± SD, *n* = 3

The ESR result revealed that the decellularization with SDS and Triton increased water uptake value compared with control, although the difference was not significant. The results also indicated that the swelling ratios increased with the increase of SDS and Triton concentration and incubation time (Fig. 6A).

The degradation results during 30 days are shown in Fig. 6B. Although the tissues denuded with SDS 0.5, 30, and ST 0.5, for 30 min showed the highest degradation rate and mas loss (%), the difference was not significant when compared with other groups (Fig. 6B).

### Morphology of microstructure of scaffold by scanning electron microscopy

**Fig. 7:**
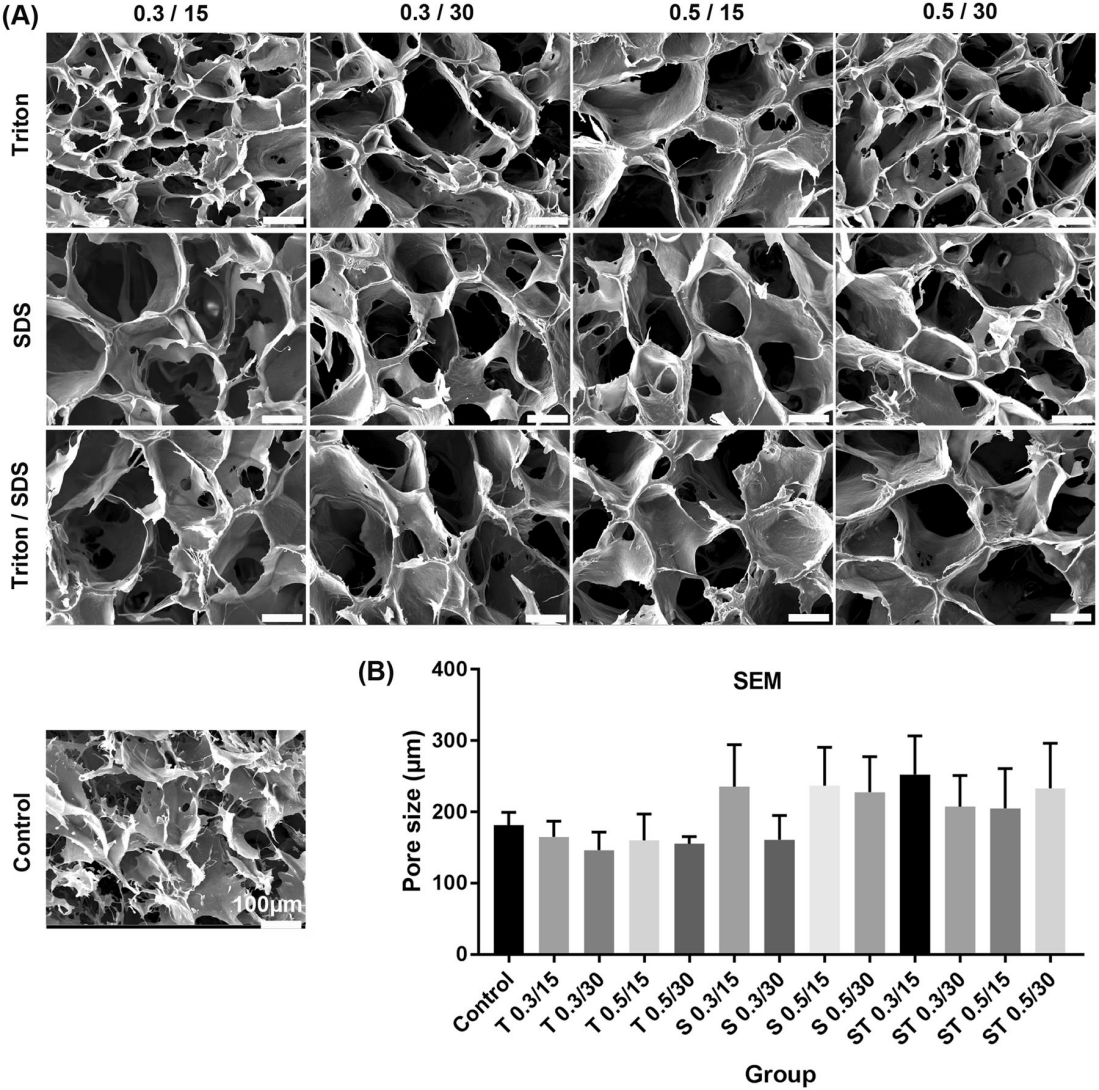
**A** The microstructure and pore size of the scaffolds. **B** Data are expressed as means ± SD, scale bar: 100 µm, *n* = 5

SEM analysis was done for assessing the microstructure and pore size of the scaffolds. The means of pore size of the scaffolds was 181.5 ± 17, 227.75 ± 49.61, 233.25 ± 63.14 in control, SDS 0.5%, 30 and ST 0.5% for 30 min groups, respectively (Fig. 7A, B).

### In vitro SSCs-scaffold interaction

#### Viability and identification of SSCs

**Fig. 8:**
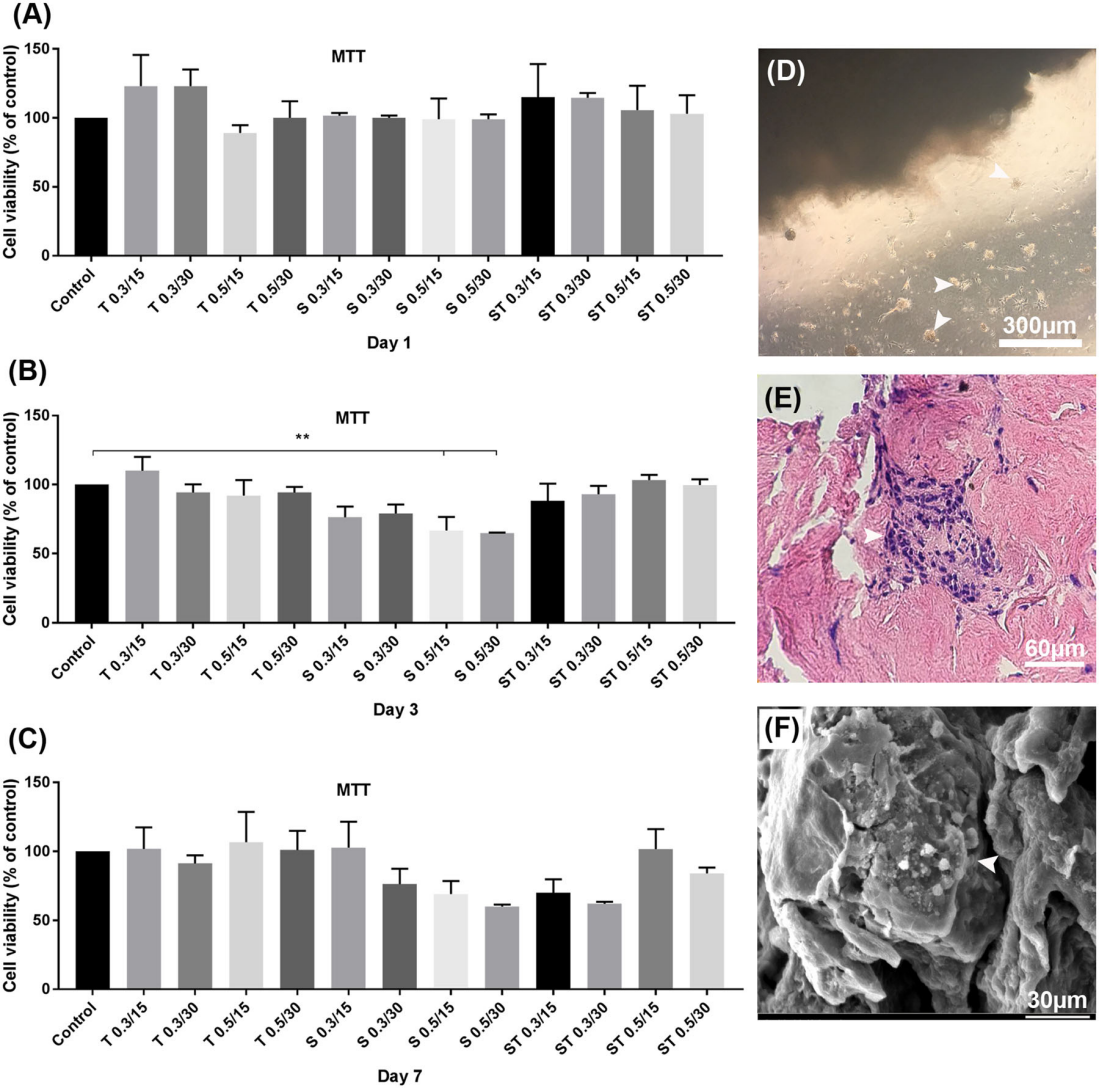
In vitro biocompatibility and bioactivity. **A**–**C** The scaffolds were placed in a 24-well plate, and seeded with 1 × 10^4^ number of SSCs. The cells-scaffold constructs were incubated in a cell culture incubator. MTT assay after 1 (**A**), 3 (**B**) and 7 (**C**) days of culture evaluated. **D** SSCs cultured indirectly in the bottom of the plates, with the presence of ST 0.5 30 scaffold and colony formation observed after 7 days. **E** H&E staining confirmed SSCs 3-D colony formation into the scaffold after 7 days. **F** SEM results confirmed SSCs 3-D colony formation onto the scaffold after 7 days. SSCs colonies are shown by white arrows. **P < 0.05. Data are expressed as means/percentage ± SD, *n* = 3

The viability of the SSCs isolated from 3 to 6 days NMRI mice was 93 ± 2%, determined by trypan blue staining. In addition, agarose gel electrophoresis confirmed the presence of mouse spermatogonial genes after two steps of mouse tastes enzymatic digestion (Fig. 8).

#### Cell proliferation and attachment

The attachment and proliferation of the SSCs cultured for 1, 3, and 7 days on all the groups of scaffolds were assessed by MTT assay. Only the placenta treated with SDS 0.5 for 15 min and 30 min showed a significant decrease in the cell viability at 3 days of culture, when compared with other groups (*P* ≤ 0.05). ST 0.5 for 30 min showed no change in cell viability in all incubation time periods of 1, 3, and 7 days (*P* ≥ 0.05, Fig. 8A–C). As only SDS 0.5/30 and ST 0.5/30 showed full removal of the cells from tissue after decellularization process, and contrary to SDS 0.5/30, ST 0.5/30 had no negative effects on SSCs viability during 7 days follow up, ST 0.5/30 was considered as optimized decellularized placental sponge, and subjected to colony formation and in vivo biocompatibility assays.

The morphology of the SSCs colony grown for 7 days on ST 0.5/30 scaffold under SEM is shown in Fig. 8F. The SSCs colony formation was also observed in the tissues stained with H&E (Fig. 8E).

### In vivo biocompatibility

**Fig. 9:**
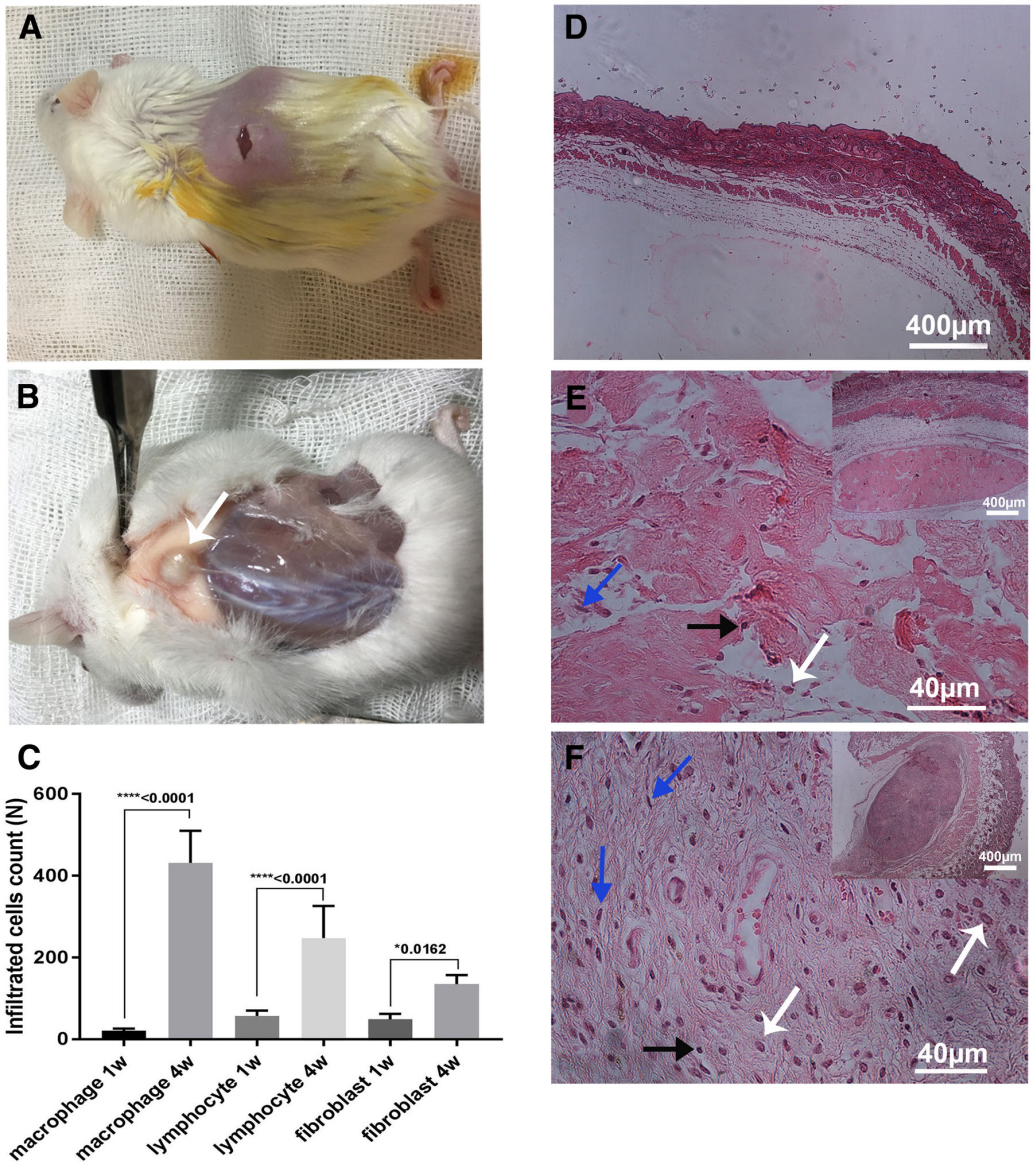
Biocompatibility of the scaffold in vivo. **A**, **B** Subcutaneous implantation of the scaffold in mouse model. After short-term (1 week) and long-term (4 week), the implanted site were collected and stained with H&E. The implanted scaffold is shown by white arrow. **C** A semi-quantitative scoring of the cells infiltrated into the implanted scaffold. The infiltrated macrophage, lymphocyte and fibroblast are shown with white, black and blue arrows, respectively. **D** H&E stained slide of normal skin with no implantation as negative control. H&E stained samples after implantation of the scaffold after short-term (1 weeks) (**E**) and long-term (4 weeks) (**F**) periods

The cells infiltrated into the subcutaneously implanted ST 0.5/30 scaffold at short-term (7 days) and long-term (30 days) post-implantation period are shown in Fig. 9c–f. The number of infiltrated macrophages increased significantly after 1 and 4 weeks, respectively (21.29 ± 4.95 to 431.9 ± 78.54, nuclei/mm^2^, mean ± SD, *P* ≤ 0.05). Also, the number of infiltrated lymphocytes increased significantly after 1 and 4 weeks (57 ± 13.11 to 247.7 ± 78.49, nuclei/mm^2^, mean ± SD, *P* ≤ 0.05) and the number of infiltrated fibroblasts increased significantly after 1 and 4 weeks (48.71 ± 13.39 to 135.4 ± 21.61, nuclei/mm^2^, mean ± SD, *P* ≤ 0.05, Fig. 9).

## Discussion

In the current study, we used different concentrations of SDS and Triton, alone and in combination, and two incubation time periods of 15 and 30 min to optimize an effective and safe method for decellularization of human placenta and fabrication of 3-D macroporous scaffold for tissue engineering and reproduction applications. As mentioned earlier in introduction, decellularization and preservation methods can remarkably affect the biomechanical and biological properties of tissue [17, 18]. Therefore, it is very important to use an optimal concentration and incubation time point of detergents with successful removal of cells and minimal negative effects on ECM proteins and growth factors [17, 29]. In our previous study, we optimized a decellularization procedure for decellularization of amniotic membrane using chemical agents such as EDTA and NaOH [28]. Placental tissue is very cellular compared amniotic/chorion membrane and need more aggressive method for successful decellularization. The ability to translocate cells and genetic material from tissue determines the importance of the widely used ionic surfactants such as SDS [30]. SDS showed to have the ability to complete cell and host DNA removal of rat forearm [31] porcine cornea [32], porcine heart valve [33], porcine kidney [34], human vein [35], porcine, and human lungs [36]. However, the reports revealed that the SDS has dose-dependent cytotoxicity effects on human cells, and is extremely destructive to tissue ECM [18, 19]. Therefore, treatment of tissue with an optimal concentration of SDS and complete elimination of SDS from tissue after denudation is extremely important. Triton X-100 is a nonionic surfactant which is widely used in cell lysis buffer, protein extraction solutions, and decellularization of tissues. Triton X-100 has less destructive effects and decellularization capacity, and also higher biocompatibility for human cells compared with SDS. Although Triton has been shown to be able to successfully remove the cells from some tissues, this nonionic agent is almost used after treatment of tissue with SDS, to promote decellularization process and also eliminate SDS during washing process [30, 37, 38].

During the evaluation period of decellularization, all of the samples were stored at -80C° until use, maximum for 1 month. It is well documented that the long-term in vitro preservation of decellularized tissues can negatively affect their biological properties [39]. Baiguera et al. decellularized Human tracheas and stored samples for one year in PBS at 4 °C. The mechanical and immunological properties of pig decellularized tracheal matrices remain unaffected by a 2-month storage in PBS. However, the scaffolds were increasingly degraded in particular of collagenous and elastic fiber structure [39].

All methods examined in our study significantly reduce DNA content and removed the cells compared with control groups. Only the tissues treated with SDS 0.5% and ST 0.5% both for 30 min showed full removal of the cells compared with other groups, confirmed by DNA count, (<50 ng/mg) H&E and DAPI staining. The optimal concentration of decellularization detergents for successful removal of the cells directly depends on tissue type. For example, 1% Triton and 1% SDS for 24 or 48 h, and 0.1% SDS for 24 h was optimal for decellularization of human testicular tissue [40] and ovarian tissues [41], respectively. Compared with Triton, SDS showed to cause extensive damage to ultrastructure and mechanical properties of tissues [38, 42]. For example, the human and porcine lungs treated with SDS showed more fibrotic structure, ECM damages, and growth factor loss compared with control (the native tissue) [30, 36]. Based on the data obtained from Alcian blue, Masson’s trichrome and orcein staining, only SDS 0.5% for 30 min and ST 0.3% for 30 min slightly decreased the ECM components of GAGs, collagen and elastin fibers, but the difference was not significant when compared with other groups.

In a study conducted by Vermeulen et al., different concentrations of SDS (0.01, 0.1, and 1%w/v) were used for decellularization of immature testicular tissue of 15 piglets. They reported that only SDS 1% caused a significant reduction in GAGs and collagen content in comparison with SDS 0.1% and SDS 0.01% groups, confirmed by Alcian blue and Masson’s trichrome staining [43]. Our results revealed that the collagen content considerably decreased in the SDS groups, while no significant reduction in collagen was observed in the placentas decellularized with all ST groups, indicating the protective effects of Triton against SDS during decellularization process. Collagen is an important component of the ECM, and our findings in consistent with previous reports indicating the dose-dependent degradation activity of the SDS for collagen fibers [44, 45]. Our data is also confirmed by the findings reported by Willemse and colleagues (2020). They used Triton alone or in combination with SDS to decellularize porcine livers. According to their report, Triton + SDS had higher rate of damages to collagen and GAGs compared with those tissues treated with Triton alone [46].

The effects of detergent concentrations and incubation time during decellularization process on mechanical property, degradation rate, swelling value, and pore size were also determined in our study.

Our investigations revealed that the mechanical properties of the decellularized placental scaffold decreased with the increase of SDS concentration and incubation time. This might be due to the destructive effects of SDS in higher dose on tissue ECM structural proteins. The same result was reported by Gilpin et al. They demonstrated a significant reduction in elastic and viscous moduli reduction with the increase of the SDS concentration and incubation time [30].

In our study, the percentage of scaffolds degradation slowly increased with increasing the incubation time, the weight of the scaffold remained intact, nearly 80 percent, following 30 days incubation. SDS 0.5, 30, and ST 0.5 for 30 min showed the maximum degradation rate because of having the highest hydrophilicity [47]. The swelling behavior of scaffolds facilitates cell migration, proliferation, and oxygen absorption and waste disposal [48]. We observed that the percentage of swelling slowly increased with the increase of the incubation time. Two groups of SDS 0.5, 30 and ST 0.5 for 30 min showed the maximum absorption of water after 72 h, about 60%, which is consistent with the similar investigations elsewhere [47]. Pore size is critical features for cell attachment, proliferation, and migration [49]. By increasing the concentration and time of detergents for decellularization, we observed an increase in the average of scaffolds pore size. The groups SDS 0.5, 30 ST 0.5 for 30 min showed the maximum pore size and minimum nuclei number, although the difference was not significant when compared with other groups.

The cytobiocompatibility of the scaffolds was determined by MTT assay. According to cell proliferation assay, a significant reduction in mitochondrial activity of SSCs, representing the cell number present at the time, was observed in the SDS 0.5/15 and SDS 0.5/30 groups after 3 days of culture. MTT results at day 7 post-culture showed that the ST 0.5 for 30 min did not change the proliferation and attachments of SSCs, confirming that the treatment of the SDS-treated samples with Triton and washing with PBS can successfully increase its cytobiocompatibility property. This finding may be due to reducing the concentration of the residual SDS to a nontoxic level and increased cells attachments to the scaffold after washing the SDS-treated tissue with Triton [30, 50]. Among the twelve decellularization procedures, only ST 0.5 for 30 min showed all the optimal characteristics together, such as successful cell removal, cytobiocompatibility for SSCs growth and colony formation and minimal damages to ECM. The SSCs colony was clearly observed after 7 days culture on the ST 0.5/30 scaffold. Both SEM micrographs and H&E stained samples showed well attachment and growth of the SSCs colony within scaffold’s pores. The in vivo examination of the optimized scaffold (ST 0.5/30) showed that the host cells were penetrated into the scaffolds’ pores with no sign of graft rejection or acute inflammatory response during short-term (7 days) and long-term (30 days) post-implantation follow up. Scaffold implantation caused the vascularization of connective tissue in the injured place and leads to the release of inflammatory responses. The number of infiltrated fibroblasts, macrophages, and lymphocytes increased significantly after one and four weeks which is consistent with the data published previously [28, 51]. The macrophages play the main role in the degradation of biological scaffolds [52]. All the data obtained from the current study revealed the successful removal of the cells from tissue after decellularization process and favorable mechanical property and cytobiocompatibility both in vitro and in vivo.

## Conclusions

According to our in vitro and in vivo investigations, the placenta tissue treated with %0.5 ST for 30 min show a successful removal of the cells with minimal negative effects on ECM components, uniform porous microstructure with interconnected network and cytobiocompatibility. The %0.5/30 ST placenta macroporous scaffold is suggested as an excellent substrate for proliferation, growth, and colony formation of spermatogonial cells.

## Supplementary information

Supplementary Figure 1

Supplementary Figure 2
