# Supplementary figures and images for: Optimization of decellularized human placental macroporous scaffolds for spermatogonial stem cells homing
Source: J Mater Sci Mater Med. 2021 Apr 23;32(5):47. doi: 10.1007/s10856-021-06517-7 (PMC8065005; doi:10.1007/s10856-021-06517-7)

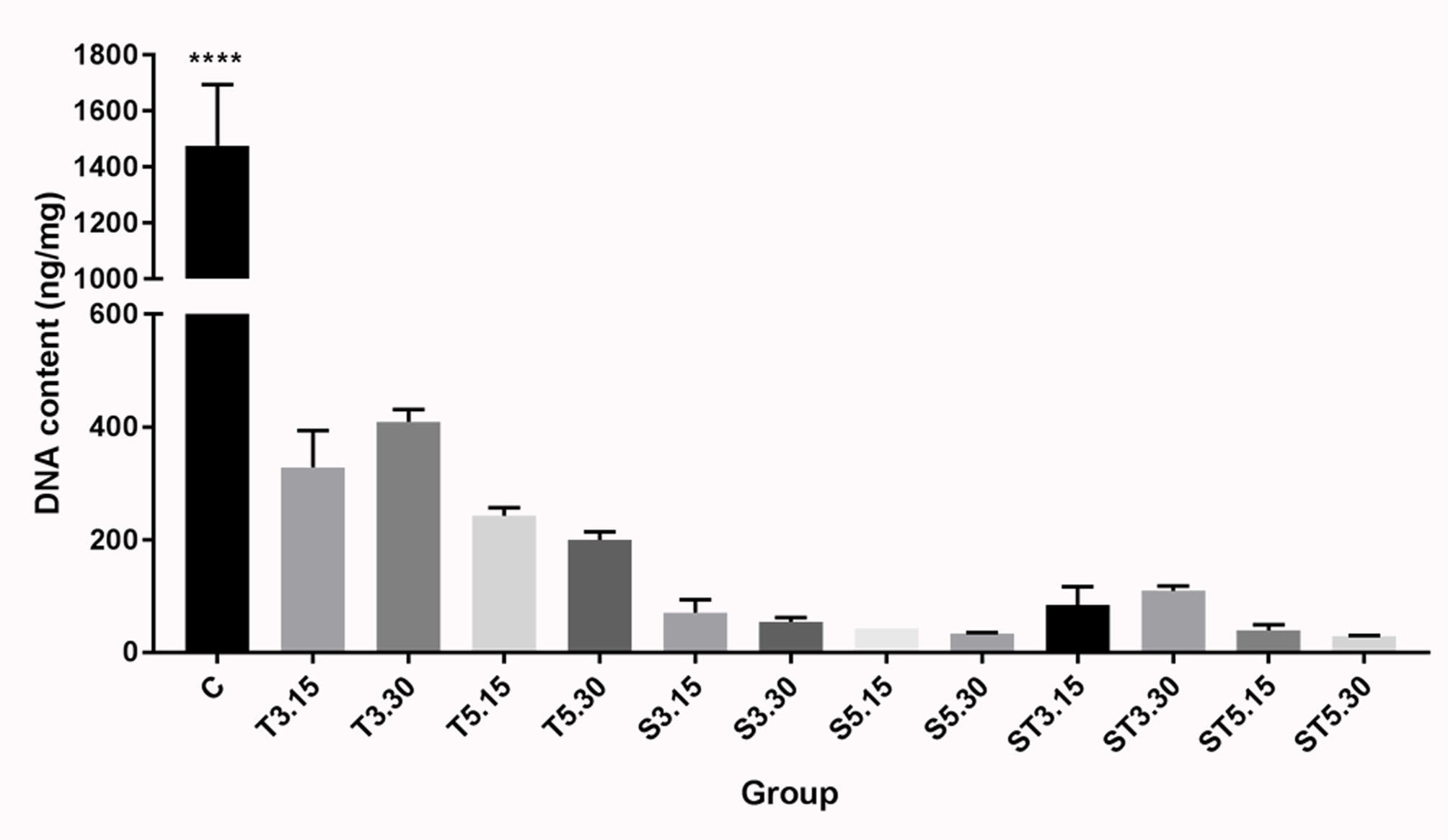

Supplement: Supplementary file 1 — Supplementary Figure 1 [file 10856_2021_6517_MOESM1_ESM.tif]

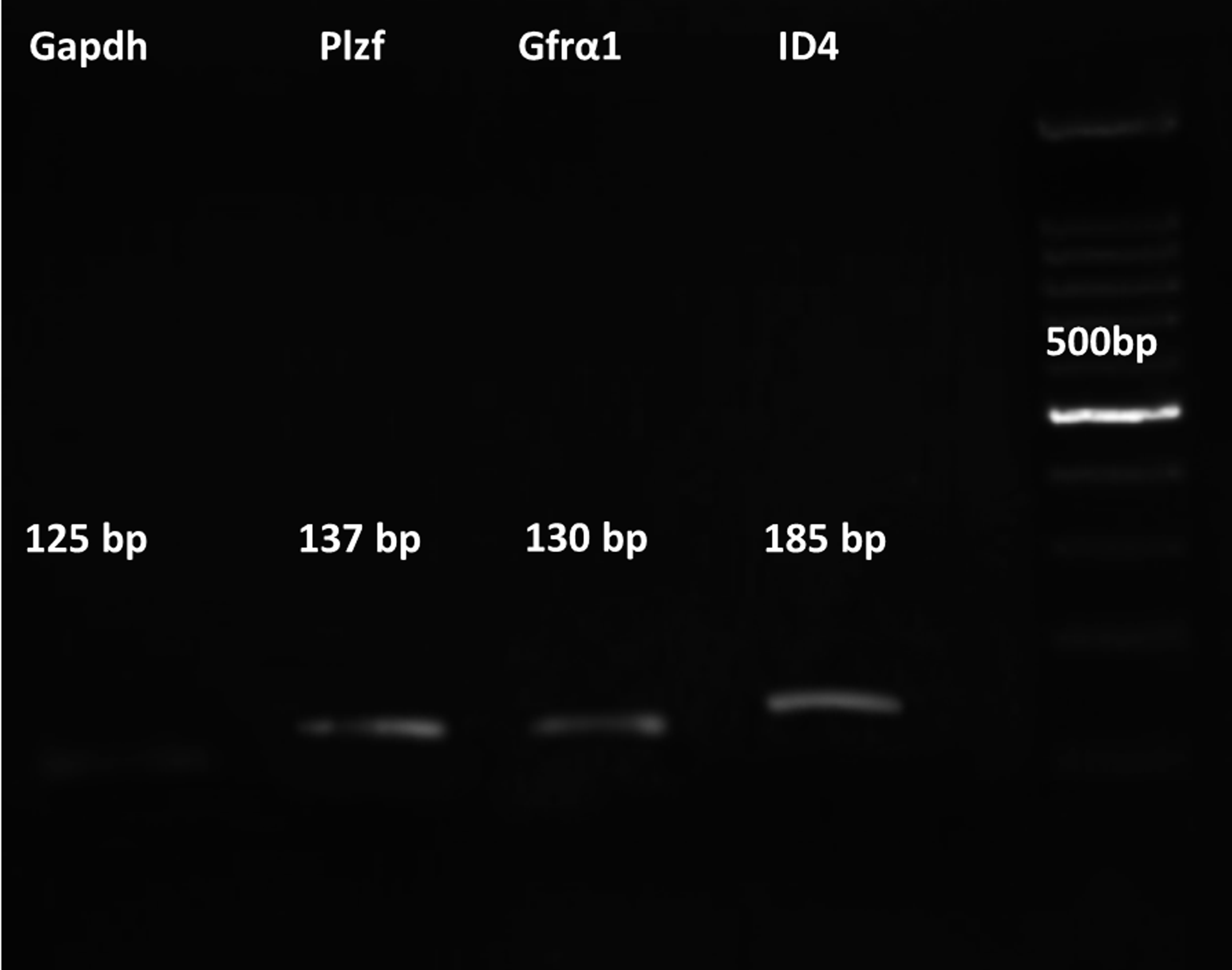

Supplement: Supplementary file 2 — Supplementary Figure 2 [file 10856_2021_6517_MOESM2_ESM.tif]
